# An Imbalance between Frequency of CD4+CD25+FOXP3+ Regulatory T Cells and CCR4+ and CCR9+ Circulating Helper T Cells Is Associated with Active Perennial Allergic Conjunctivitis

**DOI:** 10.1155/2013/919742

**Published:** 2013-12-04

**Authors:** J. Galicia-Carreón, C. Santacruz, J. Ayala-Balboa, A. Robles-Contreras, S. M. Perez-Tapia, Y. Garfias, E. Hong, M. C. Jiménez-Martínez

**Affiliations:** ^1^Department of Pharmacobiology, CINVESTAV, IPN, P.O. Box 22106, 14330 Mexico, DF, Mexico; ^2^Department of Immunology and Research Unit, Institute of Ophthalmology “Conde de Valenciana Foundation”, 06800 Mexico, DF, Mexico; ^3^Unit of R&D in Bioprocesses (UDIBI), Department of Immunology, National School of Biological Sciences, National Polytechnic Institute, 11340 Mexico, DF, Mexico; ^4^Immunology Lab, Department of Biochemistry, Faculty of Medicine, National Autonomous University of Mexico, P.O. Box 70159, 04510 Mexico, DF, Mexico

## Abstract

Allergic conjunctivitis (AC) is one of the most common eye disorders in ophthalmology. In mice models, it has been suggested that control of allergic conjunctivitis is a delicate balance between Tregs and inflammatory migrating effector cells. Our aim was to evaluate the frequency of Tregs and the frequency of homing receptors expressing cells in peripheral blood mononuclear cells (PBMC) from patients with perennial allergic conjunctivitis (PAC). The analyses of phenotypic markers on CD4+ T cells and both soluble or intracellular cytokines were performed by flow cytometry. CD4+CD25+ cells were 15 times more frequent in PBMC from patients than HC; the vast majority of these CD4+CD25+ cells were FOXP3−, and most of CD4+ T cells were CCR4+ and CCR9+ cells. Upon allergen-stimulation, no significant changes were observed in frequency of Treg; however, an increased frequency of CD4+CCR4+CCR9+ cells, CD4+CD103+ cells and CD4+CD108+ cells with increased IL-5, IL-6, and IL-8 production was observed. These findings suggest an immune dysregulation in PAC, characterized by diminished frequency of Tregs and increased frequency of circulating activated CD4+ T cells; upon allergen-stimulation, these cells were expressing cell-surface molecules related to mucosa homing and were able to trigger an inflammatory microenvironment.

## 1. Introduction

Allergies represent the most frequent chronic diseases worldwide [1]; ocular allergy is one of the most common ocular conditions encountered in clinical practice. Allergic conjunctivitis (AC) includes a spectrum of a number of traditional overlapping conditions that range from intermittent to persistent signs and symptoms, and these are fluctuating in severity and presentation. AC could be as mild forms with transient inflammation, such as seasonal (SAC) and perennial allergic conjunctivitis (PAC), or as more severe persistent and chronic inflammatory forms such as vernal keratoconjunctivitis (VKC) and atopic keratoconjunctivitis (AKC) [2, 3]. Allergic conjunctivitis is initiated by the predominant activation of CD4+ T cells to environmental allergens, culminating in a Th2 response with generation of IgE antibodies [4]. The CD4+ T cells from allergic patients are resistant to apoptosis and produce large amounts of IL-5 [5], favouring chronicity and perpetuating inflammation and relapsing-remitting symptoms. It is well known that in the chronic forms of allergic conjunctivitis CD4+ T cells are able to migrate to the ocular mucosa, maintaining the inflammatory process [6]. In mice models of ocular allergy, it has been demonstrated that CD4+CD25+FOXP3+ regulatory T cells (Tregs) influence the expression of immune-mediated allergic inflammation in conjunctiva, [7] counteracting inflammation through anti-inflammatory cytokines such as TGF-*β* and IL-10 [7, 8]. Therefore, it appears that control of allergic conjunctivitis is a delicate balance between Tregs and inflammatory migrating effector cells. In humans, intraepithelial leukocytes in the ocular surface express human mucosal lymphocyte antigen (HML or CD103) in nonpathological conditions [9], whereas in chronic allergic status, infiltrating CD4+ T cells are CCR3+ and/or CXCR3+ cells [10, 11] Nevertheless, the molecules involved in T-cell homing to conjunctiva, during the acute forms of AC in humans, are not fully studied yet. The aim of this study was to characterize the immunophenotypical features of circulating helper T cells, associated with Treg phenotype and homing receptors, in patients with perennial allergic conjunctivitis.

## 2. Methods

### 2.1. Patients

21 individuals (12 males and 9 females, mean age 11.3 years, range 5–17) with active perennial allergic conjunctivitis (PAC) were studied. Perennial allergic conjunctivitis diagnosis was based on clinical history (mean disease duration 3.5 (SD 3.1) years) and eye and physical examination. Seven healthy volunteers were used as controls (4 males and 3 females, mean age 10.2 years, range 7–15). All participants gave their informed/assent consent for blood sampling after written information was provided. The study adhered to the ethical principles of the Declaration of Helsinki, the E11 Statements of International Conference of Harmonisation (E11-ICH), and was approved by the Institutional Ethics Committee Board at the Institute of Ophthalmology “Fundación Conde de Valenciana”, Mexico City.

### 2.2. Monoclonal Antibodies and Reagents

Phycoerythrin (PE) labelled-mouse monoclonal antibodies (mAbs) against human CD25, CD103, CD108, IL-5; PECy5-labelled mAbs anti-human CD4; and fluorescein isothiocyanate (FITC)-labelled antibodies against human FOXP3 were purchased from BD PharMingen (San Jose, CA, USA). FITC-labelled mAbs anti-human CCR9 and CCR7 and PE-labelled mAbs anti-human CCR4 were from R&D Systems (Minneapolis, MN, USA). Allophycocyanin (APC)-labelled mAbs against CD4 were purchased from e-Biosciences (San Diego, CA, USA). Lymphoprep (Ficoll 1.077 density) was obtained from Nycomed Pharma (Nyegaard, Oslo, Norway). RPMI-1640 culture medium, Concanavalin A (Con A), PMA, ionomycin, saponin, brefeldin-A, and salts were from Sigma Chemical Co. (St. Louis, MO, USA). Sodium pyruvate, L-glutamine, and 2-mercaptoethanol were purchased from Gibco BRL (Rockville, MD, USA). Fetal calf serum was from HyClone Labs (Logan, UT, USA), *Dermatophagoides pteronyssinus (Der p) *was purchased from Allerstand Co. (Mexico, DF, MEX).

### 2.3. Peripheral Blood Mononuclear Cells

Whole heparinized peripheral blood was diluted 1 : 2 (vol/vol) in phosphate buffered saline (PBS), pH 7.2. Peripheral blood mononuclear cells (PBMC) were separated on a Ficoll density gradient by centrifugation at 1700 rpm for 30 min at room temperature. After centrifugation, the cells in the interface were collected, washed twice, and counted using a handheld automated cell counter (Millipore Co., Billerica, MA, USA), and viability was assessed by eosin dye exclusion.

### 2.4. Immunofluorescence Staining of Cell Surface Markers

Double or triple-colour staining was performed on PBMC by direct immunofluorescence, using APC- or PECy5-mAb anti-CD4 and either FITC- and/or PE-labelled mAbs against CD25, CD103, CD108, CCR4, CCR7, or CCR9. Briefly, 2 × 10^5^ cells were suspended in 20 *μ*L PBS supplemented with 0.2% bovine serum albumin and 0.2% sodium azide (PBA) and were incubated with fluorochrome-labelled mAb for 30 min at 4°C. After incubation, the cells were washed twice with PBA, fixed with 1% p-formaldehyde, and analysed by flow cytometry.

### 2.5. Immunofluorescence Staining of Intracellular Markers

Stimulated or nonstimulated PBMC were washed with PBA and stained with APC- or PECy5 labelled mAbs against CD4 and/or PE labelled mAbs against CD25 for 30 min. After washing, the cells were fixed with 4% p-formaldehyde in PBS for 10 min at 4°C. The cells were washed twice with PBS and permeabilised with saponin buffer (0.1% saponin and 10% BSA in PBS) by shaking gently for 10 min at room temperature. The cells were then incubated with FITC-labelled anti-human FOXP3 antibodies and/or PE-labelled anti-human IL-5. In all cases isotype-matched controls were used.

### 2.6. Cell Cultures

PBMC were cultured in 96-well flat bottomed cell culture plates (Costar, Cambridge, MA, USA) at 2 × 10^5^ cells/well in RPMI-1640 medium supplemented with 1 mM sodium pyruvate, 2 mM L-glutamine, 50 *μ*g/mL gentamicin, and 0.5% heat-inactivated fetal calf serum and incubated at 37°C in a 5% CO_2_ humidified chamber. After 24 h the culture medium was removed, and fresh culture medium supplemented with 10% heat-inactivated fetal calf serum and *Der p* (7.5 *μ*g/mL) were added. After 7 days of culture, the cells were harvested and processed to measure intracellular FOXP3 expression, and homing receptors on cell surface by flow cytometer. The Con A mitogen (2 *μ*g/mL) was used as a cell stimulation positive control. Supernatants were collected and stored at −70°C to determine soluble cytokines. In order to determine intracellular IL-5, four hours before antigen or polyclonal cultures ended, brefeldin-A was added (10 *μ*g/mL), and at the end of the incubation period the cells were harvested and were processed to immunofluorescence staining as described above.

### 2.7. Flow Cytometric Analysis

All cells were analysed for the expression of phenotypic markers on a FACScan flow cytometer (Becton Dickinson, San Jose, CA) using cell quest software, and 10000 events were counted. To analyse the staining of cell-surface markers, the lymphocytes were first gated by their physical properties (forward and side scatter), then a second gate was drawn based on immunofluorescence characteristics of the gated cells, assessing fluorescence intensity by histograms. To determine Tregs the cells were gated on FSC-SSC dot plot, then the lymphocytes were gated on CD4+ T cells in a SSC-CD4 dot plot, then CD4+ cells were selected, and a CD4-CD25 dot plot was created to select, the double positive CD4+CD25+ T cells; finally to analyse intracellular FOXP3 staining on CD4+CD25+ T cells a histogram was created to analyse the mean fluorescence intensity (MFI) of FOXP3+ cells. Data are presented as dot-plots or histograms. Control stains were performed using isotype-matched mAb of unrelated specificity. Background staining was <1% and was subtracted from experimental values.

### 2.8. Determination of Soluble Cytokines

IL-1b, IL-6, IL-8, IL-10, IL-12p70, and TNF-*α* (Human Inflammation Cytokine Kit. BD Biosciences, Franklin Lakes, NJ, USA) were measured with cytometric bead arrays (CBA) in supernatants samples according to manufacturer's instructions (BD Biosciences) and analysed by flow cytometry with BD cytometric bead array software version 1.1.1 (Becton Dickson).

### 2.9. Statistical Analysis

Mann-Whitney *U* tests or Wilcoxon Rank Signed test was used to detect significant differences. The analysis was performed with Graphpad Prism software v.5.0. Differences were considered statistically significant when the test yielded *P* values less than 0.05.

## 3. Results

### 3.1. Frequency of CD25+ Cells and CD4+CD25+FOXP3+ Regulatory T Cells in Peripheral Blood Mononuclear Cells

**Figure 1:**
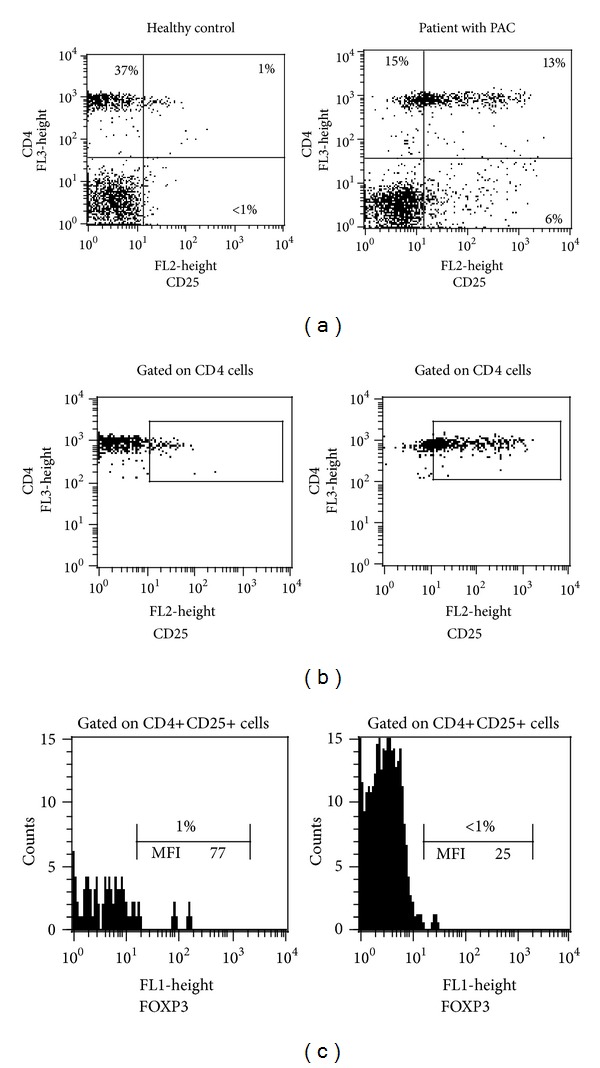
Frequency of CD25+ cells and CD4+CD25+FOXP3+ regulatory T cells in peripheral blood mononuclear cells. (a) Frequency of CD4+, CD25+, and double positive to CD4+CD25+ cells in PBMC from HC and PAC patient. (b) Same dot plot as (a); CD4+ cells gated as previously described in materials and methods, showing a gate performed to analyse CD4+CD25+ double positive cells. (c) Histogram from CD4+CD25+ gated cells (gated in (b)); the *x*-axis denotes FOXP3 (MFI = mean fluorescence intensity). These dot plots and histograms are representative of 7 HC and 14 PAC patients.

We began by determining the percentage of CD25+ and CD4+CD25+ T cells in the peripheral blood of 14 patients with allergic conjunctivitis and 7 healthy controls. As expected, the percentage of CD4+ T cells were similar among patients with perennial allergic conjunctivitis (PAC) and healthy controls (HC) (MD 27%, IQR 24–30 versus MD 34%, IQR 28–35, resp.; *P* = 0.07); meanwhile, the frequency of CD25+ cells was significantly increased in patients with PAC compared to HC (MD 5%, IQR 2.9–8.2 versus MD 0.1%, IQR 0.07–1.5, resp.; *P* = 0.001). Likewise, the percentage of CD4+ T cells expressing CD25 were also significantly increased in patients with PAC in comparison to HC (MD 18.6%, IQR 7.2–22 versus MD 0.5%, IQR 0.4–1.7, resp.; *P* = 0.0006) (Figures 1(a) and 1(b)). When we analysed the frequency of FOXP3 on CD4+CD25+ gated cells, the majority of the CD25+ helper cells were FOXP3− in both PAC and HC groups (MD 3.4% IQR 1–7 versus MD 1.8% IQR 0.4–9, resp.; *P* = 0.5) (Figure 1(c)). Interestingly, MFI in FOXP3+ cells from patients with PAC was significantly decreased when it was compared with MFI in FOXP3+ cells from HC (MFI 17 ± 9 versus MFI 93 ± 13, resp.; *P* < 0.0001) (Figure 1(c)).

### 3.2. Frequency of Chemokine Receptors on Peripheral Blood Mononuclear Cells

**Figure 2:**
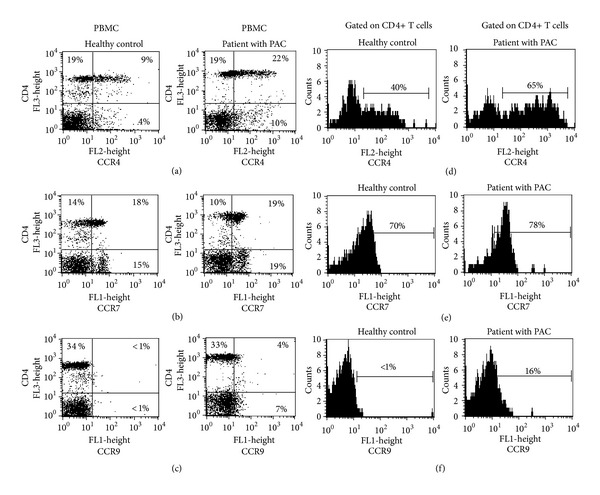
Frequency of chemokine receptors positive cells on circulating PBMC from healthy controls (HC) and from patients with perennial allergic conjunctivitis (PAC) PBMC were stained with fluorescence-conjugated antibodies to CD4 and (a) CCR4, (b) CCR7, and (c) CCR9, in a double-immunofluorescence assay, as described in materials and methods. Representative histograms of CD4+ gated cells are shown (d) CD4+CCR4+, (e) CD4+CCR7+, and (f) CD4+CCR9+ cells in both, HC and patients with PAC. Representative dot plots and histograms from 7 HC and 14 PAC.

To determine whether chemokine receptor expression was associated with a particular T helper cell traffic molecule in patients with PAC, CCR4, CCR7, and CCR9 was measured on PBMC. Results are summarized in Table 1. It was observed that CCR4+ cells were 1.9 times more frequent on PBMC from patients with PAC than in HC (*P* = 0.004). Most of the CCR4+ cells were CD4+ T cells, and CD4+CCR4+ cells were 1.8 times more frequent in patients with PAC than in HC (*P* = 0.03) (Figures 2(a) and 2(d)). We did not observe differences in frequency of CCR7+ or CCR7− cells neither on PBMC nor on CD4+ cells among groups (Figures 2(b) and 2(e)). The CCR9+ cells were 4.2 times more frequent on PBMC from patients with PAC than in HC (*P* = 0.01), and the CD4+ T cells expressing CCR9 were 2.5 times more frequent in patients with PAC than in HC (*P* = 0.01) (Figures 2(c) and 2(f)) (Table 1).

### 3.3. Frequency of CD4+CD25+FOXP3+ Regulatory T Cells and Cell-Migration Receptors after *Dermatophagoides pteronyssinus (Der p)*-Stimulation

**Figure 3:**
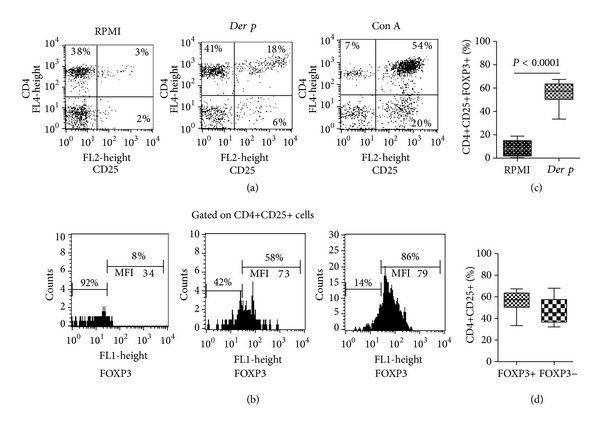
Frequency of CD25+ cells and CD4+CD25+FOXP3+ regulatory T cells after stimulation. (a) Representative dot plots of PBMC stimulated with *Der p* or Con A in patients with PAC. (b) Comparative histograms of FOXP3+ frequency on CD4+CD25+ gated cells. The *x*-axis denotes the mean fluorescence intensity (MFI) of FOXP3. (c) Significant differences were observed in the frequency of Tregs upon *Der p*-stimulation. (d) Distribution of FOXP3+ and FOXP3− cell subsets on CD4+CD25+ gated cells after allergen-stimulation.

**Figure 4:**
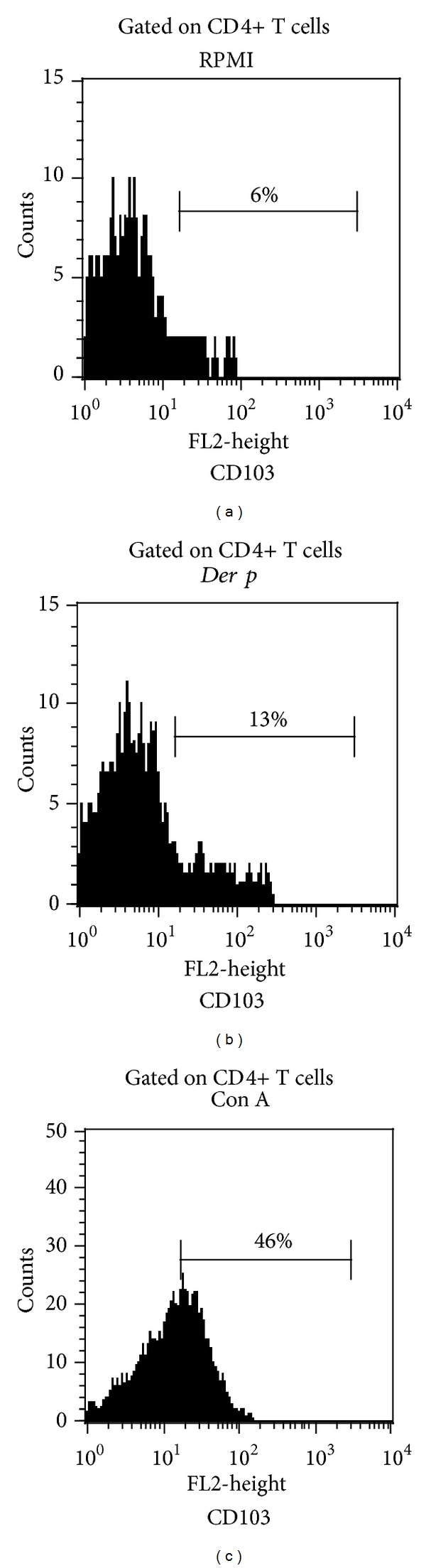
**  **Increased frequency of CD4+CD103+ T cells after allergen-stimulation. (a) RPMI (nonstimulated cells, culture medium). (b) *Der p*-stimuli. (c) Con A mitogen (Positive stimulation control); *x*-axis denotes frequency of CD103+ cells on CD4+ gated cells from patients with PAC. Representative histograms of CD4+CD103+ T cells are shown.

**Figure 5:**
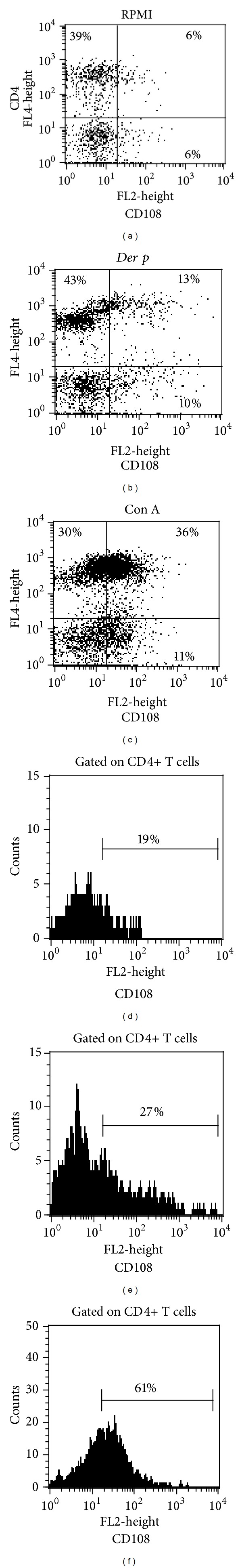
Increased frequency of CD108+ cells after allergen-stimulation. Representative dot plots of (a) nonstimulated cells, (b) *Der p*-stimulated cells, and (c) Con A-stimulated cells. Representative histograms of CD4+ gated cells, (d) nonstimulated cells, (e) *Der p*-stimulated cells, and (f) Con A-stimulated cells; *x*-axis denotes fluorescence to CD108 on CD4+ gated cells.

To establish the potential involvement of the specific antigenic-stimulation in the expression of FOXP3 and in the upregulation of cell migration receptors in PBMC from patients with PAC, we assessed the percentage of CD4, CD25, FOXP3, CCR4, CCR7, CCR9, CD103, and CD108 after *Der p* stimulation in 7 patients with active perennial allergic conjunctivitis. The specific allergic condition to *Der p* was confirmed by a skin-prick test positive to *Der p* (wheal, >3 mm diameter) and determination of IgE specific to *Der p1* (49.8 ± 39.5 kU/L). After allergen-stimulation, we observed a significant increase in the percentage of CD25+ cells (*P* = 0.0007) and CD4+CD25+ cells (*P* < 0.0001) (Figures 3(a) and 3(b)); and although frequency of CD4+CD25+FOXP3+ regulatory T cells was increased 7-folds when compared with nonstimulated cells (*P* < 0.0001) (Figure 3(c)), we did not observe significant differences between the frequency of FOXP3+ versus that of FOXP3− subsets on gated CD4+CD25+ cells after specific stimuli (Figure 3(d)). Afterwards, we analysed the frequency of chemokine receptors positive cells, and 1.9 times more CCR9+ cells (*P* = 0.04) and 2.5 times more CD4+CCR4+CCR9+ cells were observed (*P* = 0.01) after *Der p*-stimulation (Table 2). No significant changes were observed in the percentage of the following cell subsets: CCR4+, CD4+CCR4+, CCR7+, CCR7-CD4+CCR7+, and CD4+CCR7− cells. Moreover, we observed 4.1-fold more CD4+CD103+ cells (*P* = 0.007) (Figure 4), 2.5-fold more CD108+ cells (*P* = 0.01), and 4.9-fold more CD4+CD108+ cells (*P* = 0.01) after allergen specific stimulation (Figure 5). Results are summarized in Table 2.

### 3.4. Cytokines after* Der p*-Stimulation

**Figure 6:**
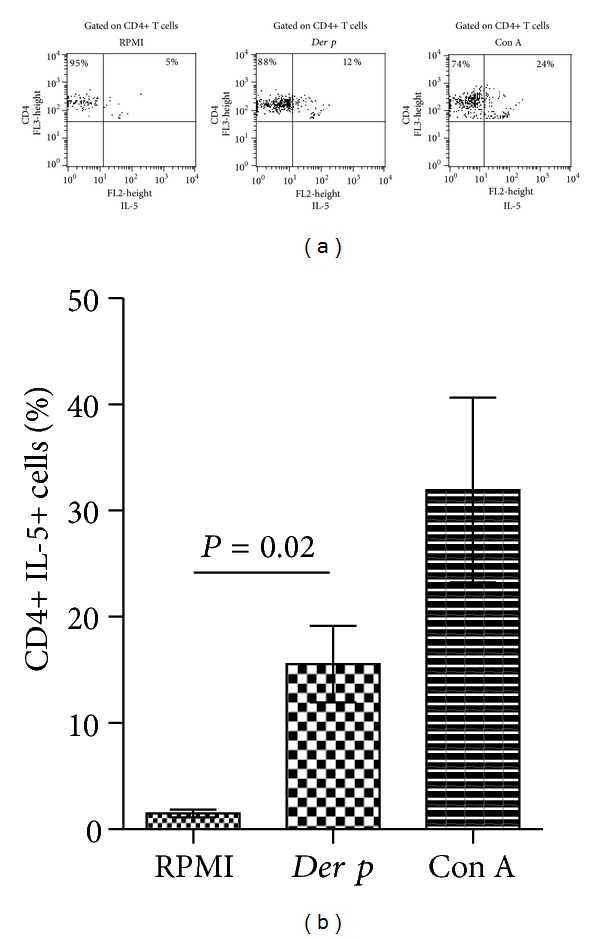
Frequency of CD4+ IL-5+ cells after *Der p*-stimulation. PBMC were *Der p*-stimulated for 72 h and stained for CD4 and intracellular IL-5 as described in materials and methods. (a) Left panel, nonstimulated cells, central panel, *Der p*-stimulated cells and right panel, and Con A-stimulated cells; representative dot plots from three PAC patients. (b) Comparison of the frequency of CD4+IL-5+ cells among different stimuli. Data are expressed as mean ± SD.

The levels of secreted cytokines IL-1b, IL-6, IL-8, IL-10, IL-12p70, and TNF-*α* were determined in culture supernatants after *Der p*-stimulation. IL-6 and IL-8 were significantly increased when compared with nonstimulated cells (*P* = 0.01 and *P* = 0.04, resp.). Results are depicted in Table 3. In order to know if *Der p-*stimulation induced early secretion of IL-5 in CD4+ cells, we performed intracellular evaluation of IL-5 in CD4+ T cells from patients with PAC. We observed 10.3 times more frequency of CD4+IL-5+ cells after *Der-p* stimulation when compared with RPMI alone (Mean 15.5% SD 3.6 versus Mean 1.4% SD 3, resp.; *P* = 0.02) (Figure 6).

## 4. Discussion

Allergic conjunctivitis is an inflammation of the conjunctiva secondary to an immune response caused by contact with an allergen at the bulbar or tarsal conjunctiva in a previously sensitized individual [12]. Two types of AC have been described, the acute forms and the chronic forms; acute forms included SAC and PAC and are the most frequent types of AC and are clinically characterized by itching, redness, and tearing [2, 3, 12, 13]; VKC and AKC are the chronic forms and they could lead to permanent visual impairment due to persistent inflammation [13, 14]. In the chronic forms of AC, allergen-mediated inflammation is maintained by infiltrating CD4+ cells to conjunctiva [15]; migration of effector cells (T cells and non-T cells) is dependent of CCR3 and CXCR3 expression [10, 11]. In acute forms of AC, molecules involved in CD4 recruitment have not been enough studied. In mice models of AC, induction of CD4+CD25+FOXP3+ regulatory T cells suppresses effector-cell activation through synthesis of IL-10 and TGF-b [7]; nonetheless, the frequency of Tregs in patients with AC has not been described yet.

In this work we analysed the frequency of circulating Tregs and the frequency of cells expressing molecules involved in T-cell homing to mucosa inflammation, in patients with perennial allergic conjunctivitis. Our results are in accordance with other authors [16–19] that have reported changes in frequency of CD4+CD25+ T cells and CD4+CD25+FOXP3+ regulatory T cells in atopic diseases, such as asthma, rhinitis, and atopic dermatitis [16–19]. These authors suggest that changes in the frequency of Tregs or impairment of their regulatory capacity could be associated with the activation of allergic status. In this work, the majority of CD4+CD25+ cells were FOXP3− cells; and after allergen-stimulation, no differences were observed among the frequency of FOXP3+ cells and FOXP3− cells; this result is relevant since it is well known that IL-6 is able to suppress Treg differentiation [20]. It is possible that after encountering with the antigen, allergen-specific CD4+ T cells from PAC patients would be able to secrete IL-6, as it was observed after *in vitro* stimulation, interfering with CD4+CD25+FOXP3+ differentiation, since it has been described that Treg cell differentiation requires antigen stimulation by engagement T cell receptor to induce FOXP3 expression [21]. Supporting this idea, the induction of FOXP3 by Con A-stimulation could be explained because of the polyclonal activation through mannose ligands on PBMC by Con A [22, 23]; nevertheless, FOXP3+ cells induced by Con A are mainly NnTregs, a different T cell subset of Tregs [24, 25].

On the other hand, it has been described that CD25 is the alpha subunit of IL2R and its expression has been related to activation status of T cells [26]; interestingly, in this study, the frequency of PBMC expressing this cell surface marker was significantly elevated in PAC patients group in contrast to healthy individuals, suggesting that PBMC and circulating CD4+ T cells are in an activation status, as similarly reported in other allergies like asthma [27, 28] rhinitis [28], and dermatitis [29].

A differential expression of chemokine receptors was observed in patients with PAC; most CD4+ T cells were CCR4+ cells, and CCR9+ cells. These findings are remarkable, as CCR4 is known to modulate T-cell migration to sites of allergic-mediated inflammation in asthma and rhinitis [30, 31]; CCR4+ cells are also an important source of IL-4 and other Th2 cytokines [30, 31]. CCR9 is a molecule expressed on antigen-experienced memory T cells and it was described as a chemokine marker related to mucosal-homing [32]. It is possible that circulating CD4+CCR4 and/or CD4+CCR9+ cells observed in PBMC from our patients are cell-subsets in transit to conjunctiva, since after allergen-stimulation increased significantly the percentage of CD4+CCR4+CCR9+ cells. Remarkably, IL-4 is required for CCR9 imprinting on CD4+ T cells [33], and it is recognized that IL-4 and IL-5 are induced after *Der p* stimulation in allergic patients and promote allergic status [5].

HML-1 or CD103 (*α*_*E*_ *β*_7_ integrin) was first described as a molecule related to mucosal migration [34], and the vast majority of intraepithelial lymphocytes are CD103+ cells [9]. HML-1 could be induced by epithelial cells on activated lymphocytes [35] and has been implicated in epithelial T-cell retention through binding to E-cadherin [36]. In the present study, upon allergen specific-stimulation it was observed an increased percentage of CD4+CD103+ T cells. The CD4+CD103+ cells have been proposed as regulatory T cell subset [37]. Likewise, after *Der p* stimulation, the percentage of CD108+ cells were significantly increased. The CD108 (Sema7a) is a glycosylphosphatidylinositol-anchored semaphorin and has been described as a molecule that initiates T-cell-mediated inflammatory response through interaction with alpha1beta1 integrin [38]. On the other hand, it has been proposed that CD108 exists as a complex with TCR and/or CD3 on T cell surface and after activation inhibits T cell signalling and decreases proliferation [39]. In this context, whether the expansion of these two subsets, CD4+CD103+ cells or CD4+CD108+ cells, is related to mucosa-homing or corresponds to regulatory T cell subsets trying to counterbalance inflammatory CCR4+CCR9+ cell subsets and IL-6/IL-8 secretion is not known and needs further investigation.

The data shown here could lead to new perspectives in the treatment of the most frequent ocular condition seen by ophthalmologists and allergo/immunologists; CCR4 and CCR9 molecules could be used as potential targets for biological therapy in patients with PAC, as it has been proposed for asthma and the monoclonal anti-CCR4 antibody [40].

Taken together, it is possible that the interaction of CCR4, CCR9, and possibly CD103 and CD108 with their ligands secreted or expressed on activated endothelial cells and conjunctiva favours the selective adhesion of a circulating CD4+ T cell subset with an activated phenotype and the ability to respond to antigens, driving the immune-response to the ocular mucosa and inducing a proinflammatory microenvironment related to Th2 perennial allergic conjunctivitis.

## Figures and Tables

**Table 1:** Frequencies of chemokine receptors in patients with PAC and HC.

Chemokine receptor	Patients with PAC	Healthy controls	*P*
	MD (IQR-Range)	MD (IQR-Range)	
CCR4+	19% (15.8–40.6)	10% (9.6–14.2)	0.004
CCR7+	35% (17–47.7)	33% (29.2–37.4)	0.688
CCR7−	65% (52.3–83)	67% (62.6–70.8)	0.688
CCR9+	18% (8–29.8)	4% (3.9–6.5)	0.01
CD4+CCR4+	42% (31.1–53.8)	23% (20.8–43.8)	0.03
CD4+CCR7+	67% (45.6–77.6)	61% (24.8–76)	0.433
CD4+CCR7−	33% (22.4–54.4)	39% (24–75.2)	0.433
CD4+CCR9+	18% (8–29.8)	4% (3.8–6.5)	0.01

MD: Median, IQR: Interquartile range.

**Table 2:** Frequencies of cell-subsets and cell-migration receptors after *Der p*-stimulation.

Cell-subsets	RPMI MD (IQR-Range)	*Der p* MD (IQR-Range)	Con A mitogen MD (IQR-Range)	*P*	
				RPMI versus *Der p *	*Der p* versus Con A
CD25+	12.5% (10.9–15.7)	20.4% (16.0–26.4)	87.5% (78.2–93.4)	0.001	<0.0001
CD4+CD25+	16.2% (12.5–20.8)	28.2% (23–32.7)	99.4% (99–99.5)	0.0004	<0.0001
CD4+CD25+ F0XP3+	3.5% (1.8–14.9)	57% (50–63.5)	18% (12.8–28.4)	<0.0001	<0.0001
CCR4+	19% (13.2–28)	21% (16–27.6)	69% (56.6–80.2)	0.312	0.002
CCR7+	55% (41.5–63.5)	49% (36–56.9)	84% (72.1–88.2)	0.687	0.0006
CCR7−	45% (36.5–58.5)	51% (43.1–64)	16% (11.8–27.9)	0.687	0.0006
CCR9+	1% (0.5–2.2)	2% (1.9–9.9)	3.4% (2.9–7.6)	0.04	0.62
CD4+CCR4+	32% (21.6–50.7)	32% (24.8–45.3)	91% (84.7–97.5)	0.62	0.001
CD4+CCR7+	86% (79.7–89.1)	82% (74.2–86.9)	98% (95.0–98.5)	0.06	0.0006
CD4+CCR7−	14% (10.9–20.3)	18% (13.1–25.8)	2% (1.5–5.0)	0.06	0.0006
CD4+CCR9+	1% (0.6–3.4)	4% (2.5–19.5)	3.7% (2.9–6.6)	0.15	0.62
CD4+CCR4+ CCR9+	5.2% (2.5–5.5)	13% (10–42.7)	6% (5.4–9.8)	0.01	0.01
CD103+	4% (2.6–7.5)	6% (4.0–7.1)	33% (26.0–34.2)	0.295	0.001
CD108+	9% (30–14.4)	23% (15.6–35.5)	76% (63.8–76.7)	0.01	0.0003
CD4+CD103+	2% (0.9–2.8)	7% (3.3–11.0)	38% (13.8–57.2)	0.007	0.001
CD4+CD108+	6% (1.3–7.6)	27% (14.5–29.9)	76% (69.7–84.8)	0.01	0.002

MD: Median; IQR: Interquartile range.

**Table 3:** Cytokines concentration in supernatants of cell culture.

	RPMI MD (IQR-Range)	*Der p* MD (IQR-Range)	Con A MD (IQR-Range)	*P*	
				RPMI versus *Der p *	*Der p* versus Con A
IL-1*β*	55.6 (50.2 ± 63.6)	52.4 (44.3 ± 67.9)	115 (96.9 ± 205)	0.99	0.01
IL-6	118.9 (72.3–291.2)	480.6 (284 ± 515.3)	2554 (1724 ± 6239)	0.01	0.001
IL-8	13579 (11145 ± 14853)	14753 (14332 ± 18661)	12657 (11094 ± 14287)	0.04	0.01
IL-10	62.51 (55.9–104.9)	69.1 (60.8–175.4)	243 (173–324)	0.32	0.09
IL-12p70	51.6 (50.5–63.9)	48.1 (46.4–49.2)	4458 (2077–6951)	0.06	0.001
TNF-*α*	34.9 (33.0–61.8)	32.3 (31.23–34.7)	6942 (4070–19610)	0.07	0.0007

MD: Median; IQR: Interquartile range.

Results are in pg/mL; kit detection limits were as follows: IL-1*β*: 3.7 pg/mL; IL-6: 4.7 pg/mL; IL-8: 3.4 pg/mL; IL-10: 4.1; IL-12p70: 4.0; TNF-*α*: 3.9 pg/mL.
